# The Added Value of Postoperative Axial Imaging in Developmental Dysplasia of the Hip

**DOI:** 10.2174/1874325001711010567

**Published:** 2017-07-26

**Authors:** Nabil Alassaf, Joud Abuhaimed, Nouf Almahmoud, Rawan Binkhulaif

**Affiliations:** 1 Department of surgical specialties, King Fahad Medical City, P.O. BOX 59046, Riyadh 11525, Kingdom of Saudi Arabia; 2 College of Medicine, Princess Nourah bint Abdulrahman University, P.O. BOX 84428, Riyadh 11671, Kingdom of Saudi Arabia

**Keywords:** Hip joint, Hip dysplasia, Hip dislocation, Radiography, Computed tomography, Redislocation

## Abstract

**Background:**

Redislocation is a dreaded complication after reduction of developmental dysplasia of the hip (DDH) in young children. While early detection facilitates urgent reoperation, delayed revisions are more complicated. Despite the weak evidence, an axial postoperative imaging tool is recommended. This study’s goal is to compare the effectiveness of conventional pelvic radiography alone and axial imaging.

**Methods:**

Data were collected retrospectively between 2012 and 2016. One study group comprised consecutive patients who had operative reduction followed by routine low-dose computed tomography (CT). Hips that had anteroposterior pelvic radiographs as the only confirmatory tool were used as a reference group.

**Results:**

We identified 241 patients (339 hips). The mean age and follow up were 19.6 months ± 9.3, and 15.5 months ± 11.1, respectively. There were 147 hips in the radiography group and 192 in the CT group. Radiography detected only three out of nine redislocations during the same admission; in contrast, 2/2 redislocations in the routine CT group were addressed before hospital discharge (p<0.01). There was no significant delay in hospital discharge when CT was used (p= 0.28).

**Conclusion:**

Conventional radiography is not as effective as axial imaging in preventing late detection of redislocation.

**Level of Evidence:**

level III, Diagnostic Study.

## INTRODUCTION

Late diagnosis of developmental dysplasia of the hip (DDH) is a major health problem in some parts of the world, and leads to a high number of operative interventions [1]. It is generally accepted that children who fail splinting and those older than six months are treated in the operating room under general anesthetic with either closed reduction (CR) or open reduction (OR). The latter is performed if there is obstructing tissue or if the children are older than two years to facilitate simultaneous pelvic osteotomy, but some authors recommend forgoing CR after the age of 18 months [2, 3]. Femoral shortening osteotomy is added based on intraoperative assessment to relocate the femoral head without excessive pressure [4].

A dynamic hip arthrogram is used to confirm the adequacy of reduction in DDH intraoperatively. However, after the cast application and when the anesthetic wears off, redislocation may occur. Therefore, confirmatory images are obtained in the immediate postoperative period. Several diagnostic tests are used, namely conventional anteroposterior pelvic radiographs, ultrasound through a window in the cast, computed tomography (CT), and magnetic resonance imaging (MRI) [5-7].

Anteroposterior pelvic radiograph is less favored postoperatively for theoretical reasons; Superimposed plaster material often precludes adequate visualization of the hip after reduction. Samuelson *et al.* used conventional tomography so no redislocation went unnoticed [7]. A few years later, many centers reported the use of CT to assess reduction [8, 9]. Although visualization may appear adequate, posterior redislocation may be difficult to recognize with the hip flexed and abducted in the “human frog” position [10]. Eggli *et al.* found that low-dose CT, equivalent radiation of 0.64 mSv, is as diagnostic as higher doses CT when used post-DDH reduction [11]. A recent study estimated total dose to be 0.5 mSV [12]. That is about six times more radiation compared to conventional chest radiographs [13]. The dose varies according to patient size and gender for the same exposure. Ionizing radiation in children is linked to an increased risk of cancer later in life, but the long-term effect of pediatric low-dose pelvic CT has yet to be determined [14-16].

To avoid ionizing radiation, magnetic resonant imaging (MRI) was introduced for use in DDH patients. It was later found to be as effective as CT in confirming concentric reduction, and because the child is immobilised in a cast, general anaesthetic is seldom needed [17]. CT is more cumbersome than conventional radiography, and inconvenient for the patients and their families, and occasionally sedation is required, which is not the case for conventional radiographs. Research examining the effectiveness of radiography alone compared to 3D assessment tools in detecting early redislocation is scarce. The main goal of this study is to evaluate whether conventional pelvic radiography can substitute for three-dimensional imaging in the timely detection of redislocation after operative reduction of DDH. The secondary objective is to quantify the hospital stay among patients who have had CT compared to conventional pelvic radiographs.

## MATERAL AND METHODS

### Patient Selection

**Fig. (1):**
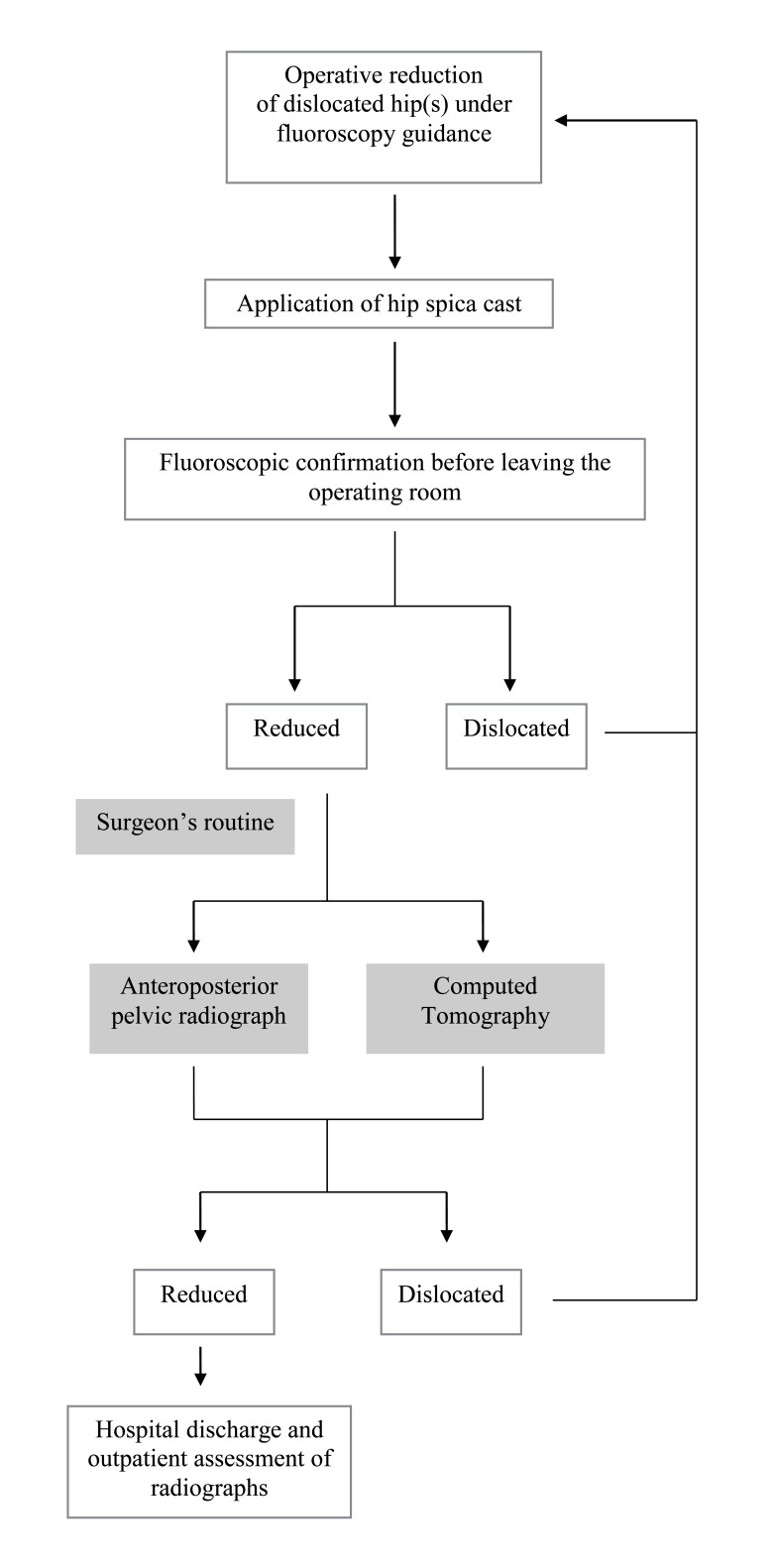
Imaging pathway.

We obtained approval from the local ethics committee of our institution (approval number 15-359). A retrospective data collection design was used. We performed a census of all DDH patients who were operated on between September 2012 and June 2016. This multi-surgeon cohort included two groups according to the attending physicians’ routine practice (Fig. **1**). The CT group is patients who had undergone routine immediate post-reduction low-dose pelvic CT prior to hospital discharge. The second group, the radiography group, had undergone conventional anteroposterior pelvic radiograph. Inclusion criteria were: patients less than four years of age, no prior hip operation, International Hip Dysplasia Institute (IHDI) grade 2-4, minimum follow up of two months [18]. We excluded patients with underlying neuromuscular conditions and hips that had been stabilized with Krischner wires.

### Treatment Pathway

All the surgeries were performed under general anesthesia using routine techniques for CR and OR. All patients over one year of age had capsular plication after reduction through an anterior approach, whereas younger patients underwent medial open reduction [3, 19]. All the operated hips were immobilized in a spica cast for six to ten weeks after OR, and 12 to 16 weeks after CR. The hip was kept flexed and abducted after CR, relatively extended and abducted after OR. Postoperative images were performed within 48 hours and prior to patient discharge. The CT was not repeated afterwards. Fluoroscopy and radiographs were used during cast change and in the clinic visits.

### Outcome Parameters

Besides demographic variables, surgical details, type of post-reduction images, duration of hospital stay, complications during hospitalization including unplanned trips to the operating room for redislocation, readmissions, follow-up duration and the final Shenton’s line alignment, were collected.

### Statistical Methods

Fisher’s exact test was used for the count data. Normality of continuous data was assessed visually and found to be nonparametric, therefore the Mann-Whitney-Wilcoxon test was then applied. Two-tailed p values of <0.05 were considered to be significant. We used R software for statistical analysis, version 3.3.1 (R Foundation for Statistical Computing, Vienna, Austria).

## RESULTS

339 hips were analyzed in 241 patients. The mean (± SD) age was 19.6 months ± 9.3 (range:1 - 48). The mean follow-up was 15.5 months ± 11.1 (2 - 48). There were 206 females and 35 males. 156 hips were on the right side, and 183 on the left. 147 hips were in the radiography group and192 in the CT group. Baseline variables were balanced between the two groups, but the CT group had a higher proportion of closed reduction and lower rate of pelvic osteotomies Table **1**.

**Fig. (2):**
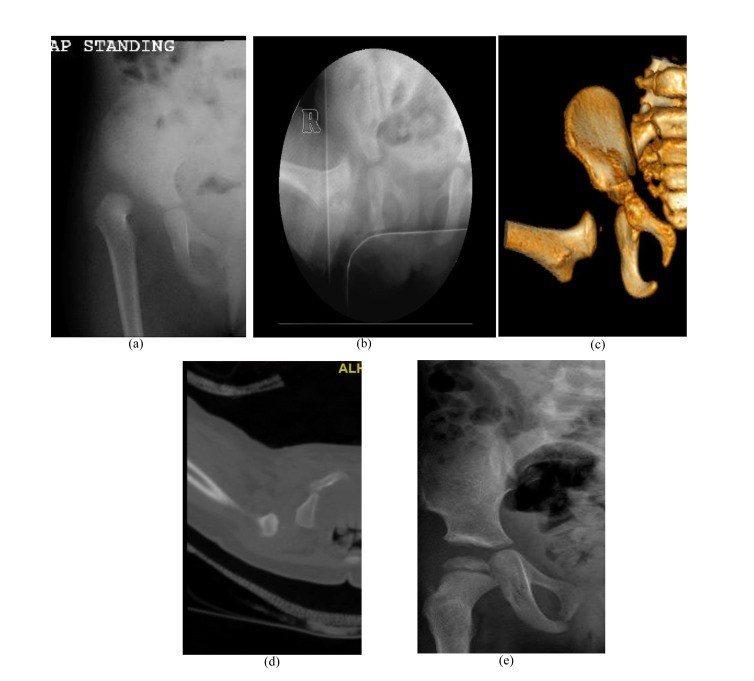
Right hip dislocation in an 18-month old girl. (a) Preoperative radiograph. (b) Intraoperative radiograph after closed reduction and cast application. (c) Frontal 3D CT-reconstructed images. (d) Axial cut showing the posterior dislocation. (e) Follow-up radiograph 22 months after salvage with open reduction and Dega osteotomy.

**Fig. (3):**
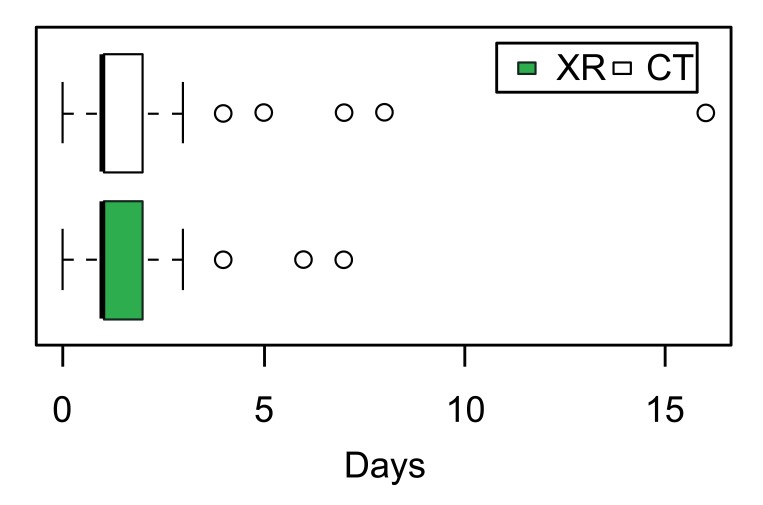
Postoperative hospital stay. XR, radiography group; CT, Computed tomography group.

Overall, redislocation was detected in 3% of this sample during the study period. Seven hips redislocated after CR (Fig. **2**), the remaining four were after OR. There was more late detection and readmission for redislocation in the radiography group (Table **2**). The rate of resubluxation within the follow-up period as defined by the disrupted Shenton’s line was not significantly different. The mean hospital stay for the radiography group was 1.4 days ±1.1 (0 - 7) and 1.5 days ±1.19 (0 - 16) for the CT group (Fig. **3**). The difference in hospital stay was not significant (p = 0.28). Of the 144 CT studies completed, 24 patients (16.7%) received Chloral Hydrate oral sedation.

Early postoperative complications that confounded duration of hospital stay were recorded in 19 patients (5.6%), upper respiratory tract infection being the most common (Table **3**). The longest hospital stay was 16 days; the patient was two years and eight months old, underwent unilateral OR and pelvic osteotomy, had persistent fever due to streptococcal septicemia, and was kept in the hospital for intravenous therapy. The infection was believed to have come from tonsillitis just before the surgery. One patient had femoral nerve transection and subsequent subluxation. The operative report indicated that the exposure was medial to the Sartorius muscle, which is a locally accepted practice.

## DISCUSSION

In this study, we provide data to support the notion that postoperative axial images are effective in excluding immediate redislocation after operative reduction of DDH. Although the overall incidence of redislocation is relatively low, the difference in early detection of displacement between the radiography group and the CT group was significant (p < 0.01). At our institution, CT is a feasible method and does not significantly prolong hospital stay. Furthermore, while conventional radiographs are usually obtained immediately after the patient leaves the operating room, CT is typically performed the following day. We do not perform post-reduction ultrasound, and are moving toward MRI as the newer machines produce less noise and the scans are performed more easily without sedation. Moreover, to reduce radiation exposure, we now, tend to do less post-reduction CT for children with largely ossified femoral heads as we believe displacement can be detected by comparing the intraoperative with the postoperative radiographs (Fig. **4**).

Most of the relevant published work lacks direct comparison that justifies the need for advanced imaging postoperatively. The reported incidence of redislocation of DDH in the literature is 0% to 20% [20-24]. Here, the redislocation rate is 3% for the two groups combined. Eberhardt *et al.* compared post-reduction standard radiographs with ultrasound in 33 hips. In two cases, the radiographs showed symmetrical femoral head location, but the ultrasound detected posterior dislocation [6]. Other studies are limited to small case series. Stanton *et al.* reviewed the scans of 42 patients over five years to determine the usefulness of CT in confirming reduction. They only included patients who had closed reduction before the age of 18 months; most patients had three studies; CT immediately after anesthesia, two weeks postoperatively and 6-8 weeks later after cast change. The CT detected two redislocations: one in the immediate postoperative CT and the other two weeks postoperatively [20]. Toby *et al.* reported their experience in 15 patients, and found unsatisfactory reduction detected in three patients by postoperative CT [21].

Not surprisingly, more interest is now in MRI. Chin *et al.* compared the accuracy of MRI and CT for hip reduction in 39 patients. The calculated CT dose in their study was only 1 mSv, the sensitivity and specificity of both studies were excellent, 8/44 hips had a redislocation [24]. Later, Sachleben *et al.* reported a lower radiation dose of 0.5 mSv [12]. The minimum follow-up was two months, which we believe is sufficient to answer our main research question. Moreover, Case *et al.* reported no redislocation after the first eight weeks following reduction in 67 hips, and the rate of redislocation was eight times higher after CR compared to OR [22]. In our study, the groups were imbalanced as there was more CR in the CT group, but this did not adversely affect the incidence of redislocation.

A strength of this study is the large homogeneous number of patients, which includes a comparative group that did not receive postoperative 3D studies. We are not aware of similar published studies. Limitations of this study include retrospective data collection, but using a prospective design is not feasible because of the low occurrence of redislocation. A more optimal study design would have been comparing both imaging modalities on the same cohort, but this would entail unnecessary imaging and more radiation exposure. Therefore a differential verification approach was used based on clinical follow up. The benefits of early detection of redislocation are efficient patient care and less complicated revision by avoiding scar tissue, but these advantages remain theoretical.

## CONCLUSION

Routine conventional pelvic radiographs do not substitute for 3D imaging in the timely detection of redislocation after operative reduction of DDH. Such confirmatory studies did not prolong hospital stay. Further research in selective imaging is suggested to minimize ionizing radiation and cost.

## Figures and Tables

**Fig. (4a):**
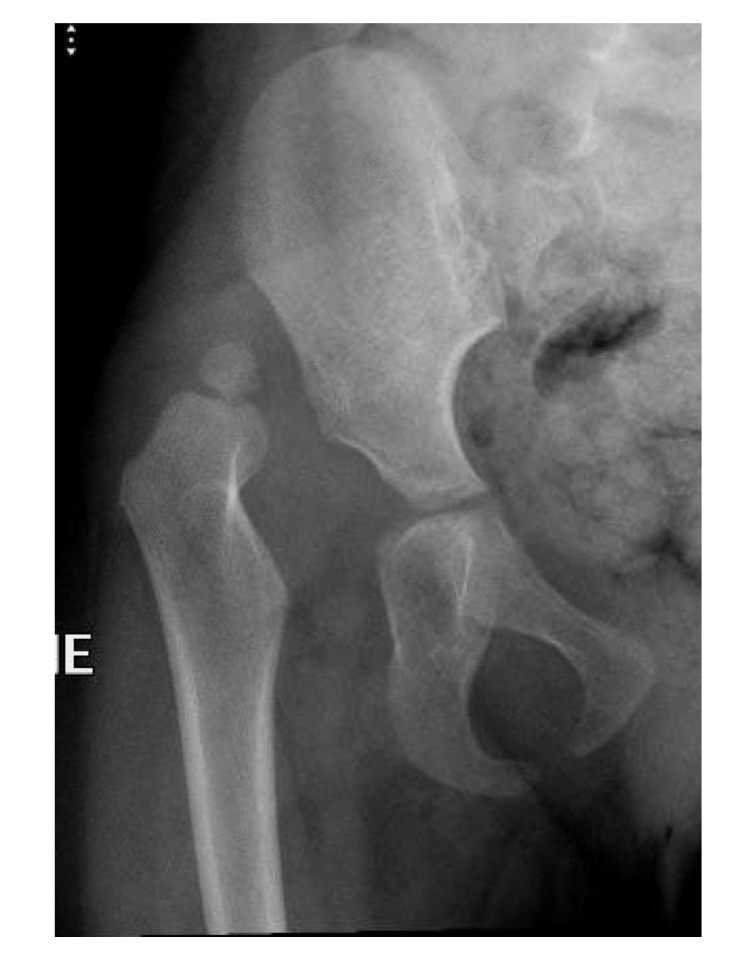
Plain radiograph of a girl at age 2 years and 9 months.

**Fig. (4b):**
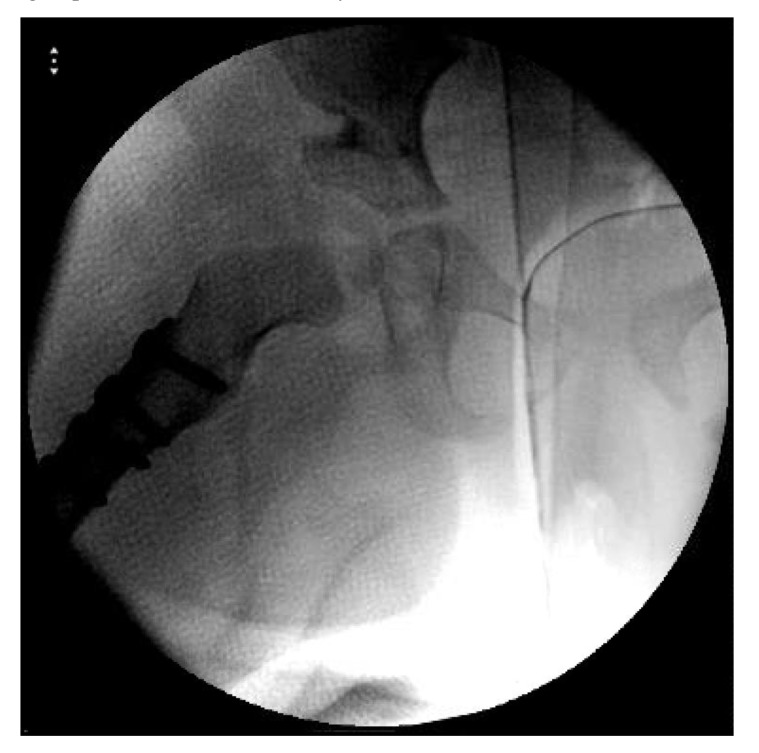
Intraoperative fluoroscopy picture after open reduction, femoral shortening and Dega osteotomy.

**Fig. (4c):**
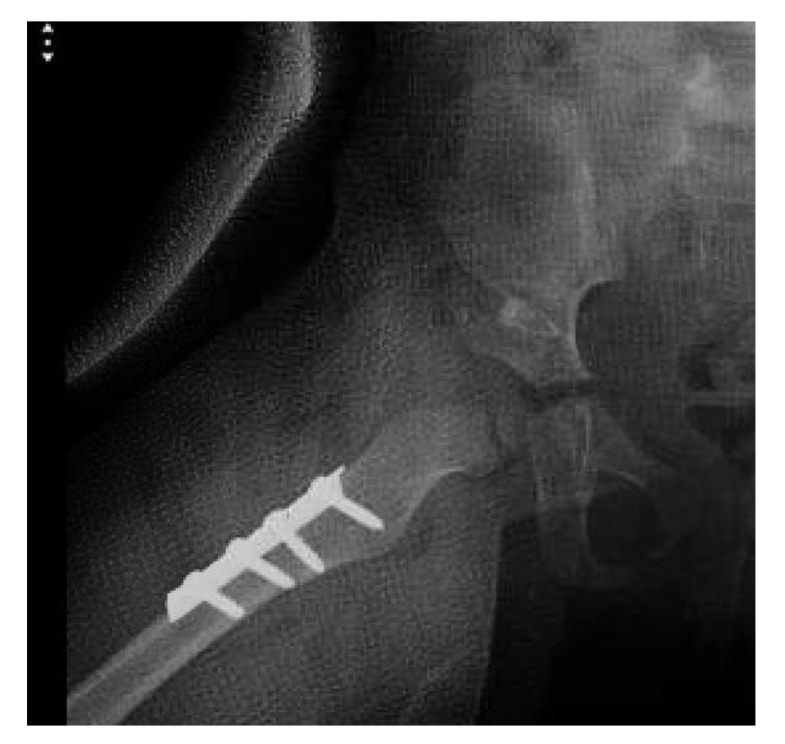
Postoperative radiograph indicating concentric reduction. Note the overlap between the ossific nucleus and the Ischium.

**Fig. (4d):**
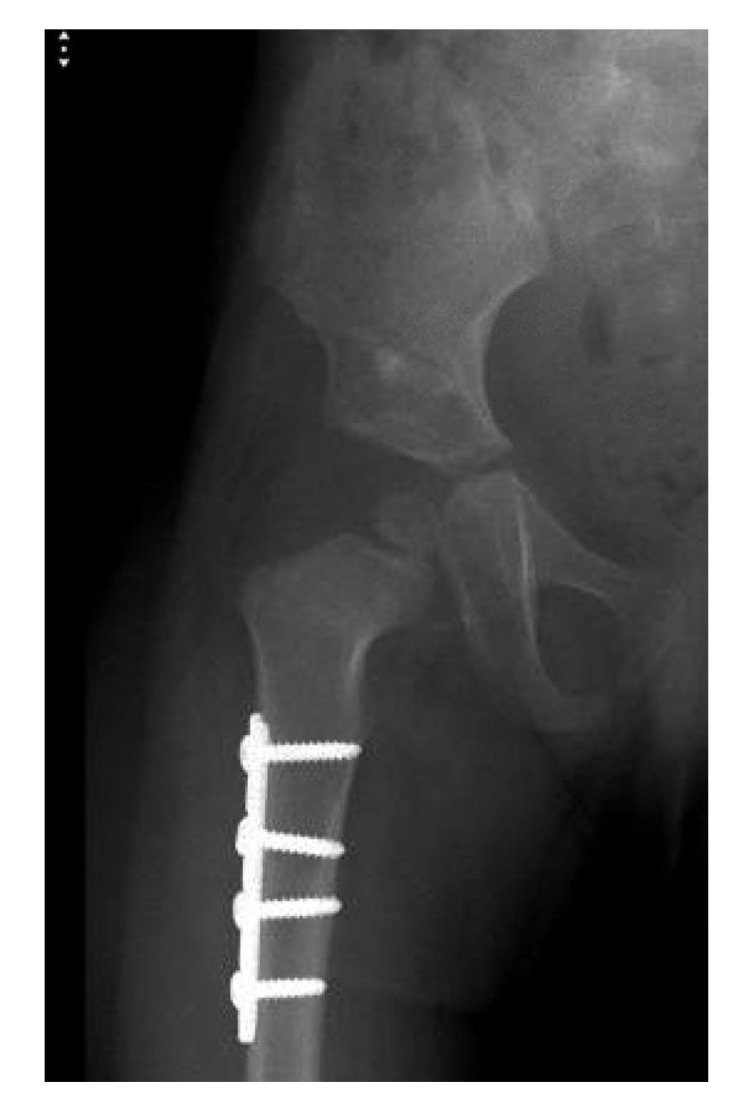
Four months postoperative radiograph showing intact Shenton line.

**Table 1:** Baseline characteristics.

	Radiography group (n=147)	CT group (n=192)
Age (months), SD, Range	20.61±10.31(range: 1-48)	18.81±8.41 (range: 2-46)
Sex F	129 (88%)	163 (85%)
M	18 (12%)	29 (15%)
Side R	67 (46%)	89 (46%)
L	80 (54%)	103 (54%)
IHDI 2	15 (10%)	16 (8%)
3	33 (22%)	53 (28%)
4	99 (68%)	123 (64%)
Reduction closed	37 (25%)	108 (56%)
open	110 (75%)	84 (44%)
Femoral shortening Yes	12 (8%)	18 (9%)
No	135 (92%)	174 (91%)
Pelvic osteotomy Yes	104 (71%)	75 (39%)
No	43 (29%)	117 (61%)
Follow-up (months), SD	15.23±12.00 (range: 2-48)	15.68±10.63 (range: 3-46)

**Table 2:** Results.

Event	Radiography group (n=147)	CT group (n=192)	Odds Ratio	95% CI	p=value
Total redislocations	9 (6%)	2 (1%)	0.16	0.02 to 0.80	0.01
Redislocations detected during admission	3 (2%)	2 (1%)	0.51	0.04 to 4.48	0.66
Redislocations detected after discharge	6 (4%)	0	0	0.00 to 0.64	<0.01
Hips with disrupted final Shenton’s line	5 (3%)	1 (0.5%)	0.15	0.01 to 1.36	0.09

**Table 3:** Complications detected during index hospitalization.

Event	Radiography group (n=147)	CT group (n=192)
Early redislocation	3 (2%)	2 (1%)
Upper respiratory tract infection	3 (2%)	6 (3%)
Otitis media	0	1 (0.5%)
Intravenous line infection	0	1 (0.5%)
Gastroenteritis	0	1 (0.5%)
Urinary tract infection	1 (0.7%)	0
Septicaemia	0	1 (0.5%)
Femoral nerve injury	1 (0.7%)	0
Total	8 (5.4%)	12 (6.2%)
